# Evaluation of bioactive extracellular soluble Hemarina (M101) derived scaffolds for neovascularization in periodontal tissue regeneration

**DOI:** 10.1016/j.jobcr.2026.01.003

**Published:** 2026-01-13

**Authors:** A. Mohamed Thaha, Kaarthikeyan Gurumoorthy

**Affiliations:** Department of Periodontics, Saveetha Dental College and Hospitals, Saveetha Institute of Medical and Technical Sciences (SIMATS), Saveetha University, Chennai, India

**Keywords:** M101, Angiogenesis, Periodontal regeneration, Chitosan–alginate scaffolds, Hypoxia, CAM assay, VEGF

## Abstract

**Background:**

Periodontitis leads to progressive destruction of periodontal tissues, where low oxygen levels and inadequate vascularization limit regenerative healing. Hemarina-101 (M101), a marine-sourced extracellular hemoglobin with high oxygen-binding and antioxidant potential, may overcome these barriers. This study investigated the ability of M101-incorporated chitosan–alginate scaffolds to support angiogenesis and cell compatibility for potential use in periodontal regeneration.

**Methods:**

Chitosan–alginate scaffolds containing 10 % w/w M101 were fabricated and compared with plain scaffolds, untreated controls, and vascular endothelial growth factor (VEGF)-treated groups. Human periodontal ligament cells (PDLCs) and endothelial cells (EA.hy926) were cultured on the scaffolds. Cell viability and proliferation were analyzed using MTT assays and live/dead staining. Expression of angiogenic genes (VEGF-A, ANGPT1, CD31, HIF-1α) was quantified by qPCR. In vivo-like angiogenic responses were assessed using the chorioallantoic membrane (CAM) assay. Statistical significance was set at *p* < 0.05.

**Results:**

Scaffolds with M101 showed significantly greater cell viability and proliferation than plain scaffolds (*p* < 0.05), comparable to VEGF-treated controls. Live/dead staining confirmed high densities of viable cells on M101 scaffolds. Gene expression analysis revealed notable upregulation of VEGF-A, ANGPT1, CD31, and HIF-1α in the M101 group, approaching levels seen with VEGF treatment. The CAM assay demonstrated dense, radially organized vessel networks forming around M101 scaffolds, indicating a strong pro-angiogenic effect.

**Conclusion:**

M101-incorporated scaffolds enhanced endothelial cell growth, angiogenic gene activation, and neovascularization compared with plain scaffolds. These findings support the potential of M101-based biomaterials as promising candidates for periodontal tissue regeneration, meriting further preclinical and clinical validation.

## 1 Introduction

Periodontitis is a chronic, inflammatory condition caused by microbial imbalance, leading to the progressive breakdown of the gingiva, periodontal ligament, and alveolar bone (Pihlstrom et al., 2005). Clinically, the disease presents as gingival bleeding, periodontal pocket formation, clinical attachment loss, abscesses, and eventual tooth loss, thereby compromising both oral function and esthetics.^1^

**Fig. 1:**
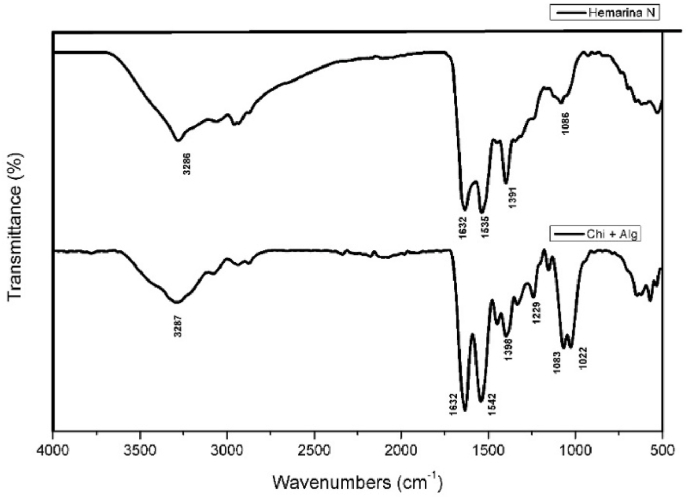
Fourier Transform Infrared (FTIR) spectra of Hemarina N and Chitosan–Alginate (Chi + Alg) scaffolds.

**Fig. 2:**
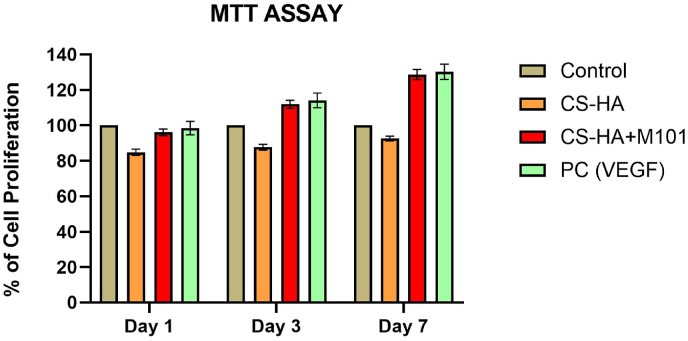
MTT assay showing the percentage of cell proliferation in different treatment groups across Day 1, Day 3, and Day 7.

**Fig. 3:**
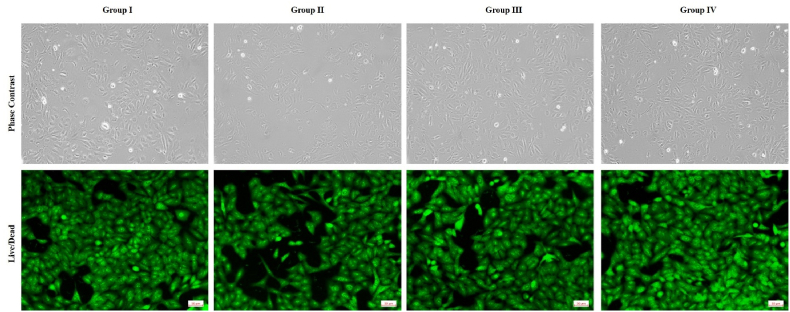
Phase contrast and Calcein-AM fluorescent microscopy analysis of cell viability and morphology on different scaffold groups.

**Fig. 4:**
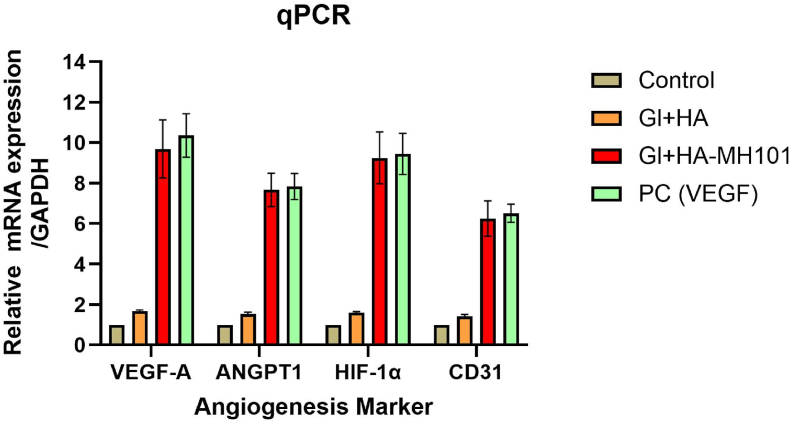
Quantitative PCR analysis of angiogenic marker gene expression in different treatment groups.

**Fig. 5:**
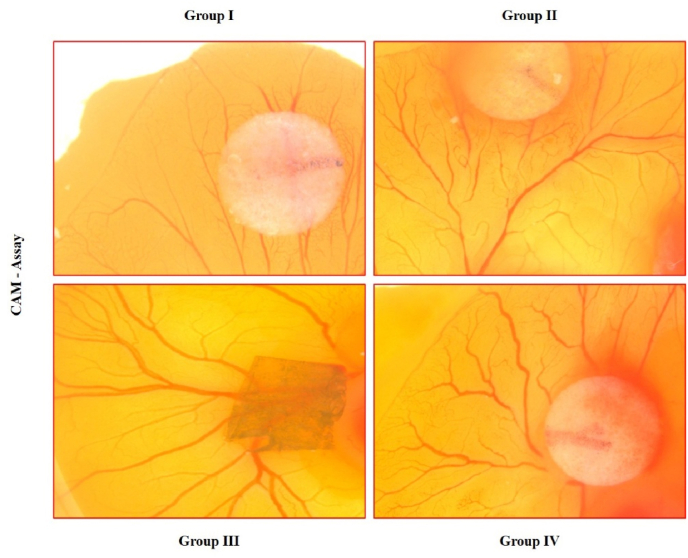
Chorioallantoic membrane (CAM) assay for neovascularization induced by M101-Infused chitosan-hydroxyapatite scaffold.

The development of periodontal regenerative therapies aims to restore the supporting periodontal structures in teeth with deep pockets and diminished periodontal support.^2^ The primary objectives of such treatments are:

1. To promote new attachment formation and bone regeneration around severely affected teeth.
2. To achieve reduced probing pocket depths (PPD) for long-term periodontal stability.
3. To minimize or prevent gingival recession to maintain esthetics.^2^

Regeneration techniques have proven effective in managing intrabony defects of varying wall configurations (one-, two-, and three-wall defects or combinations) regardless of their size or morphology. The success of surgical therapy largely depends on the creation of a wound environment that fosters optimal healing, enabling the reconstruction of the dentogingival complex and facilitating improved oral hygiene practices.^3^

Wang and Boyapati (2006) introduced the PASS principle, comprising Primary wound closure, Angiogenesis, Space maintenance, and Stability of the wound clot, as a biological framework for predictable tissue regeneration. This principle advocates the use of barrier membranes and grafts to prevent the rapid down growth of the epithelium, thereby enabling regenerative cells to repopulate the defect site. The concept has been successfully adapted in Guided Bone Regeneration (GBR) for implant-related procedures as well.^4^

A wide range of grafts, barrier membranes, and biomaterials have been investigated for periodontal regeneration. Although no single material has demonstrated absolute superiority, studies indicate that combinational approaches often produce better outcomes compared to using individual materials alone.^5^

Recent advances have introduced haemoglobin-based oxygen carriers to enhance tissue oxygenation during regeneration. Traditional formulations using human or bovine haemoglobin failed in later clinical trials due to safety limitations. In contrast, the biotech company HEMARINA has developed M101, a natural extracellular haemoglobin derived from the marine worm *Arenicola marina*. M101 exhibits unique properties, including:

• The ability to carry up to 156 molecules of oxygen (compared to only 4 in human haemoglobin).
• Sustained oxygen release according to physiological gradients.
• Antioxidant activity and superoxide dismutase (SOD)-like properties that minimize ischemia-induced oxidative damage and free radical formation.^6^

HEMARINA has formulated M101 within a hyaluronic acid (HA) and xanthan gum (Xn)-based gel specifically for periodontal applications. This biomaterial ensures controlled oxygen delivery, promotes angiogenesis, and prevents oxidative stress, thereby creating a favorable healing environment. Preclinical studies have already demonstrated M101's ability to reduce ischemia/reperfusion injury in organs such as the kidney, liver, heart, pancreas, and lungs.^7^

Despite promising preclinical results demonstrating M101's protective effects against ischemia–reperfusion injury in multiple organs, its angiogenic and regenerative capabilities within periodontal contexts remain unexplored. This study aims to investigate the neovascularization potential of scaffolds infused with M101, evaluating their suitability for enhancing periodontal regeneration and facilitating defect healing.

## 2 Materials

### 2.1 Experimental groups and scaffold preparation

#### 2.1.1 Four experimental groups were established for comparative evaluation

(1) **Untreated Control (UC) –** cells without scaffold exposure, serving as a baseline reference;
(2) **Plain Scaffold (PS) –** chitosan–alginate hydrogel scaffold without bioactive incorporation;
(3) **M101 Scaffold (M101) –** PS scaffold incorporated with 10 % w/w Hemarina-101 (M101), a marine-derived extracellular haemoglobin; and
(4) **Positive Control (PC) –** cells treated with vascular endothelial growth factor (VEGF, 10 ng/mL).

Chitosan (medium molecular weight, 85 % deacetylated) and sodium alginate were dissolved separately in 1 % acetic acid and deionized water, respectively, and blended in a 3:2 ratio. M101 was incorporated into the hydrogel blend before ionic crosslinking using 2 % CaCl_2_. The scaffolds were moulded, lyophilized, and sterilized using UV irradiation prior to cellular and in vivo applications.

**Table:** 

Group	Description
**Untreated Control (UC)**	Cells without scaffold
**Plain Scaffold (PS)**	Chitosan–alginate scaffold without M101
**M101 Scaffold (M101)**	Scaffold with 10 % w/w M101 incorporation
**Positive Control (PC)**	VEGF-treated group (10 ng/mL)

#### 2.1.2 Scaffold characterization

**FTIR spectroscopy** was performed to analyze functional groups and confirm successful incorporation of M101. The spectra of plain and M101-loaded scaffolds were recorded in the range of 4000–500 cm^−1^. Characteristic peaks for chitosan and alginate (e.g., O–H, COO^−^, C–O–C vibrations) were observed in both groups. M101 scaffolds showed additional amide I and II peaks, confirming protein incorporation (Fig. 1).

#### 2.1.3 Cell culture and viability assays

Human periodontal ligament cells (PDLCs) and human umbilical vein endothelial cells (HUVECs) were cultured in DMEM supplemented with 10 % FBS and 1 % penicillin-streptomycin. For the MTT assay, cells were seeded at a density of 1 × 10^4^ cells/well in 96-well plates and exposed to the respective treatment groups for 24, 48, and 72 h. MTT reagent (0.5 mg/mL) was added and incubated for 4 h at 37 °C. Formazan crystals were dissolved in DMSO, and absorbance was recorded at 570 nm. Results were normalized and expressed as % cell viability relative to control (Fig. 2.

#### 2.1.4 Live/dead cell viability assessment

Calcein-AM and Ethidium Homodimer-1 staining was performed to evaluate live/dead cell distribution on scaffolds. After 72 h incubation with PDLCs and HUVECs on scaffold materials, samples were washed with PBS and stained with 2 μM calcein-AM and 4 μM EthD-1 for 30 min at 37 °C. Fluorescence images were captured using an inverted fluorescence microscope. Green fluorescence indicated viable cells, while red fluorescence denoted dead cells (Fig. 3).

#### 2.1.5 Gene expression analysis by qPCR

Quantitative PCR was performed to assess osteogenic and angiogenic gene expression. Total RNA was extracted using TRIzol reagent, and cDNA synthesis was carried out using a reverse transcription kit. Real-time PCR was performed using SYBR Green Master Mix and gene-specific primers for VEGF-A, ANGPT1, HIF-1α, and CD31 with GAPDH as the housekeeping gene. Relative gene expression was analyzed by the 2^−ΔΔCt method. Samples were collected at Days 7 post-treatment (Fig. 4).

#### 2.1.6 CAM assay for angiogenesis

Fertilized chick eggs were incubated at 37 °C with 65 % humidity, and a window was created on Day 7 to access the chorioallantoic membrane (CAM). Sterile scaffold samples from each group were placed directly onto the CAM, and the windows were sealed. On Day 8, the CAMs were photographed, and neovascularization was quantified by counting the number of branching vessels around the scaffold area. All experiments were conducted in triplicates (Fig. 5).

## 3 Results and interpretation

### 3.1 Scaffold preparation & characterisation

The FTIR spectrum of Hemarina N shows characteristic protein peaks, including a broad O–H/N–H stretching vibration at 3286 cm^−1^ and amide I and II bands at 1652 cm^−1^ and 1535 cm^−1^, respectively. In contrast, the Chi + Alg scaffold spectrum exhibits typical polysaccharide bands such as the O–H stretching at 3287 cm^−1^, asymmetric COO^−^ stretching at 1642 cm^−1^, symmetric COO^−^ stretching at 1398 cm^−1^, and C–O–C vibrations at 1222 cm^−1^ and 1022 cm^−1^, confirming the cross-linking of chitosan and alginate.

FTIR analysis confirmed the successful incorporation and interaction of biomolecular components in the Hemarina N protein and Chi + Alg scaffold system. The Hemarina N spectrum displayed prominent peaks corresponding to amide I (C=O stretching) at 1652 cm^−1^ and amide II (N–H bending and C–N stretching) at 1535 cm^−1^, indicative of its proteinaceous nature. Additionally, the broad absorption at 3286 cm^−1^ represents overlapping O–H and N–H stretching vibrations. The Chi + Alg spectrum demonstrated key functional groups of polysaccharides, including broad O–H stretching at 3287 cm^−1^, characteristic asymmetric and symmetric COO^−^ stretching at 1642 cm^−1^ and 1398 cm^−1^, and C–O–C stretching at 1222 cm^−1^ and 1022 cm^−1^. The retention of these functional peaks suggests successful scaffold formation and potential hydrogen bonding or electrostatic interaction sites for Hemarina-101 loading, which could enhance biological activity and scaffold biofunctionality.

### 3.2 Cytocompatibility and proliferation

**MTT Assay**: The M101-derived scaffolds supported high cell viability and proliferation rates for both EA.hy926 and PDLCs, indicating good biocompatibility.

Human periodontal ligament cells were treated with (i) Control (untreated), (ii) CS–alginate scaffold, (iii) CS–alginate incorporated with M101 (10 % w/w), and (iv) Positive Control (VEGF, 10 ng/mL). Cell proliferation was assessed at three time points—Day 1, Day 3, and Day 7—using the MTT assay. Results are expressed as % cell proliferation relative to the control group. The CS–Alg + M101 and VEGF-treated groups showed a significant increase in cell viability over time, with the highest proliferation observed on Day 7, indicating enhanced cytocompatibility and bioactivity. Error bars represent mean ± SD from three independent experiments.

### 3.3 Live/dead staining

Showed a high percentage of live cells and uniform distribution across the scaffold surfaces.

Representative microscopic images of human endothelial cells (HUVEC) cultured on different scaffold groups:

(i) Control (untreated cells without scaffold),
(ii) CS-Alg (chitosan–alginate scaffold),
(iii) CS-Alg + M101 (scaffold incorporated with M101, 10 % w/w), and
(iv) Positive Control (PC; VEGF-treated cells, 10 ng/mL).
  (A) Phase contrast images (magnification: × 100) show cell morphology and attachment patterns. Enhanced cell spreading and confluency were observed in CS-Alginate + M101 and VEGF-treated groups.
  (B) Calcein-AM staining images (fluorescence microscopy; green fluorescence) indicate viable cells, with brighter and denser fluorescence in CS-Alginate + M101 and VEGF-treated groups, suggesting improved cell viability and proliferation.

Images were captured on Day 3 of culture. The data supports enhanced cytocompatibility and pro-angiogenic potential of M101-incorporated scaffolds.

### 3.4 Gene expression by qPCR

**Angiogenic Marker Expression**: qPCR results showed significantly upregulated expression of VEGF in EA.hy926 cultured on the M101-derived scaffolds compared to controls.

Relative mRNA expression levels of VEGF-A, ANGPT1, HIF-1α, and CD31 were quantified by qPCR and normalized to GAPDH as a housekeeping gene on day 7. The study compared four groups: (i) Control (untreated), (ii) CS + Alginate (chitosan–alginate scaffold), (iii) CS + Alginate-MH101 (scaffold incorporated with M101, 10 % w/w), and (iv) Positive Control (PC; VEGF-treated group, 10 ng/mL). Notably, the CS + Alginate-MH101 and VEGF groups showed significantly upregulated expression of all four angiogenic markers, with VEGF-A and HIF-1α expression reaching the highest levels. This suggests that M101 incorporation effectively enhances angiogenic gene expression, comparable to exogenous VEGF treatment. Data represent mean ± SD from triplicate experiments.

### 3.5 CAM assay

Representative images from CAM assay at Day 8 post-implantation.

• **Group I (UC)**: Untreated control showed sparse and randomly oriented vessels with minimal branching.
• **Group II (CS-Alginate)**: Chitosan-Alginate scaffold induced moderate vessel ingrowth with some radial branching.
• **Group III (CS-Alginate + M101)**: M101-infused scaffold exhibited robust angiogenic response with dense, radiating microvascular branches converging towards the implant site.
• **Group IV (PC – VEGF)**: VEGF-treated group demonstrated intense angiogenesis and dense capillary plexus formation, confirming pro-angiogenic validation.
  Red border delineates the area of scaffold placement or angiogenic induction.

## 4 Discussion

The current study evaluated the capacity of scaffolds infused with M101—a marine-derived extracellular hemoglobin—to stimulate angiogenesis in the context of periodontal regeneration. The results complement and extend compelling preclinical findings, reinforcing the potential of M101-based biomaterials in overcoming hypoxia and inflammation within periodontal lesions.

Our findings demonstrated that scaffolds loaded with 10 % w/w M101 exhibited significantly enhanced cell viability, upregulated angiogenic marker expression, and robust neovascularization in the CAM assay compared with plain scaffolds, closely approximating the effects of VEGF treatment.

The cytocompatibility results from MTT assays revealed consistently high viability of both PDLCs and endothelial cells on M101 scaffolds over seven days, with proliferation levels approaching those observed in VEGF-treated positive controls. Live/dead staining confirmed dense, viable cell distribution, indicating a favorable microenvironment for cellular attachment and growth. These results are in agreement with *Batool* et al. (2020), who reported that M101 supports cellular bioenergetics under hypoxic conditions by delivering oxygen efficiently while minimizing oxidative stress.^7^

A significant finding was the upregulation of angiogenic genes—VEGF-A, ANGPT1, CD31, and HIF-1α—in cells cultured on M101 scaffolds. The VEGF-A expression levels in particular were comparable to those in the VEGF-treated group, demonstrating M101's potential to stimulate pro-angiogenic signaling. These findings align with *Le Meur* et al. (2021), who showed that M101 reduces ischemia–reperfusion injury by mitigating oxidative damage and promoting vascular repair.^8^

The CAM assay provided in vivo-like validation, where M101 scaffolds induced dense, radially oriented neovessels converging at the implantation site. Similar vascular patterns have been reported by *Rouwkema and Khademhosseini* (2016), who emphasized the importance of prevascularization strategies in tissue-engineered scaffolds to enhance integration and survival post-implantation.^9^

Comparatively, plain chitosan–alginate scaffolds in our study exhibited only moderate angiogenesis, consistent with previous findings that polysaccharide-based hydrogels provide structural support but lack intrinsic angiogenic stimulation unless combined with bioactive agents. By contrast, M101 incorporation appears to overcome this limitation by enhancing oxygen availability and reducing hypoxia-induced cell death—key barriers in periodontal defect healing.

Furthermore, the oxygen-carrying capacity of M101 (able to bind up to 156 O_2_ molecules versus 4 in human hemoglobin) likely contributed to the observed cellular and molecular responses. This is supported by *Zal* et al. (2017), who highlighted M101's therapeutic oxygen delivery properties in organ preservation models. Its superoxide dismutase-like activity may also explain the reduced oxidative stress and improved angiogenic outcomes observed in our study.^10^

When compared with other regenerative approaches, such as growth factor delivery systems (e.g., VEGF, FGF-2) or stem cell–laden scaffolds, the M101-based system offers distinct advantages. Growth factors often face limitations related to short half-life, high cost, and potential for uncontrolled angiogenesis, whereas M101 provides a sustained, gradient-driven oxygen supply with inherent antioxidative properties, reducing the need for supraphysiologic exogenous growth factor doses.^11^

However, some limitations must be acknowledged. Our experiments were confined to in vitro conditions and the CAM assay, which, although valuable, cannot fully replicate the complex periodontal niche involving immune–microbial interactions and mechanical loading. Moreover, the long-term stability and degradation kinetics of M101 within scaffolds remain unexplored. Future studies should employ preclinical periodontitis models to evaluate functional outcomes such as bone regeneration, inflammatory modulation, and integration with host vasculature.

In summary, the present findings, supported by existing literature, suggest that M101-incorporated scaffolds create a pro-angiogenic, cytocompatible microenvironment conducive to periodontal regeneration. By addressing the dual challenges of hypoxia and oxidative stress, this approach represents a promising alternative or adjunct to conventional growth factor–based therapies.

## 5 Conclusion

In summary, M101-infused HA–Xn scaffolds represent a compelling advancement in periodontal regenerative strategies. By simultaneously mitigating hypoxia and oxidative stress, promoting vascularization, and stabilizing the wound environment, this biomaterial addresses critical barriers in periodontal healing. Future efforts should emphasize quantitative angiogenic assessments, extended preclinical validation, and scaffold optimization—bringing this novel approach closer to clinical implementation.

## Patient consent

Nil. (Inviro-Study).

## Ethical clearance

Nil (In-vitro Study).

## Source of funding

Self Funded

## Declaration of competing interest

The authors declare that they have no known competing financial interests or personal relationships that could have appeared to influence the work reported in this paper.
